# Supplementary material for: Fragment-sequencing unveils local tissue microenvironments at single-cell resolution
Source: Nat Commun. 2023 Nov 27;14:7775. doi: 10.1038/s41467-023-43005-8 (PMC10681997; doi:10.1038/s41467-023-43005-8)
Supplement: Supplementary file 1 — Supplementary Information [file 41467_2023_43005_MOESM1_ESM.pdf]

Supplementary Information

Fragment-sequencing unveils local tissue microenvironments at single-cell resolution

Kristina Handler[1], Karsten Bach[1], Costanza Borrelli[1], Salvatore Piscuoglio[2,3], Xenia Ficht[1], Ilhan E. Acar[1] and Andreas E. Moor[1,4]

[1] Department of Biosystems Science and Engineering, ETH Zürich, Schanzenstrasse 44, 4056 Basel, Switzerland

[2] Institute of Medical Genetics and Pathology, University Hospital Basel, Basel, Switzerland,

[3] Visceral Surgery and Precision Medicine Research laboratory, Department of Biomedicine, University of Basel, Basel, Switzerland

[4] Corresponding author, andreas.moor@bsse.ethz.ch

Supplementary file legends

1. SupplementaryData_MULTI-seq_3Prime_sequences: This file contains the primer sequences used for labeling of cells following the MULTI-seq method and a 3' scRNA-seq capture approach. The first column shows the well position within 3x96 well plates; the second column shows the name of the barcode; the third column represents the barcode sequence and the fourth column shows the oligo sequence which contains a primer sequence for lipid anchor binding and amplification (CCTTGGCACCCGAGAATTCCA), an 8 bp barcode sequence and a poly-A stretch for single-cell RNA-seq capture.

2. SupplementaryData_MULTI-seq_5Prime_sequences: This file contains the primer sequences used for labeling of cells following the MULTI-seq method and a 5' scRNA-seq capture approach.
The first column shows the well position within 2x96 well plates; the second column shows the name of the barcode; the third column represents the barcode sequence and the forth column shows the oligo sequence which contains a primer sequence for lipid anchor binding and amplification (CCTTGGCACCCGAGAATTCCA), an 8 bp barcode sequence and a 5' capture sequence (CCCATATAAGAAA).

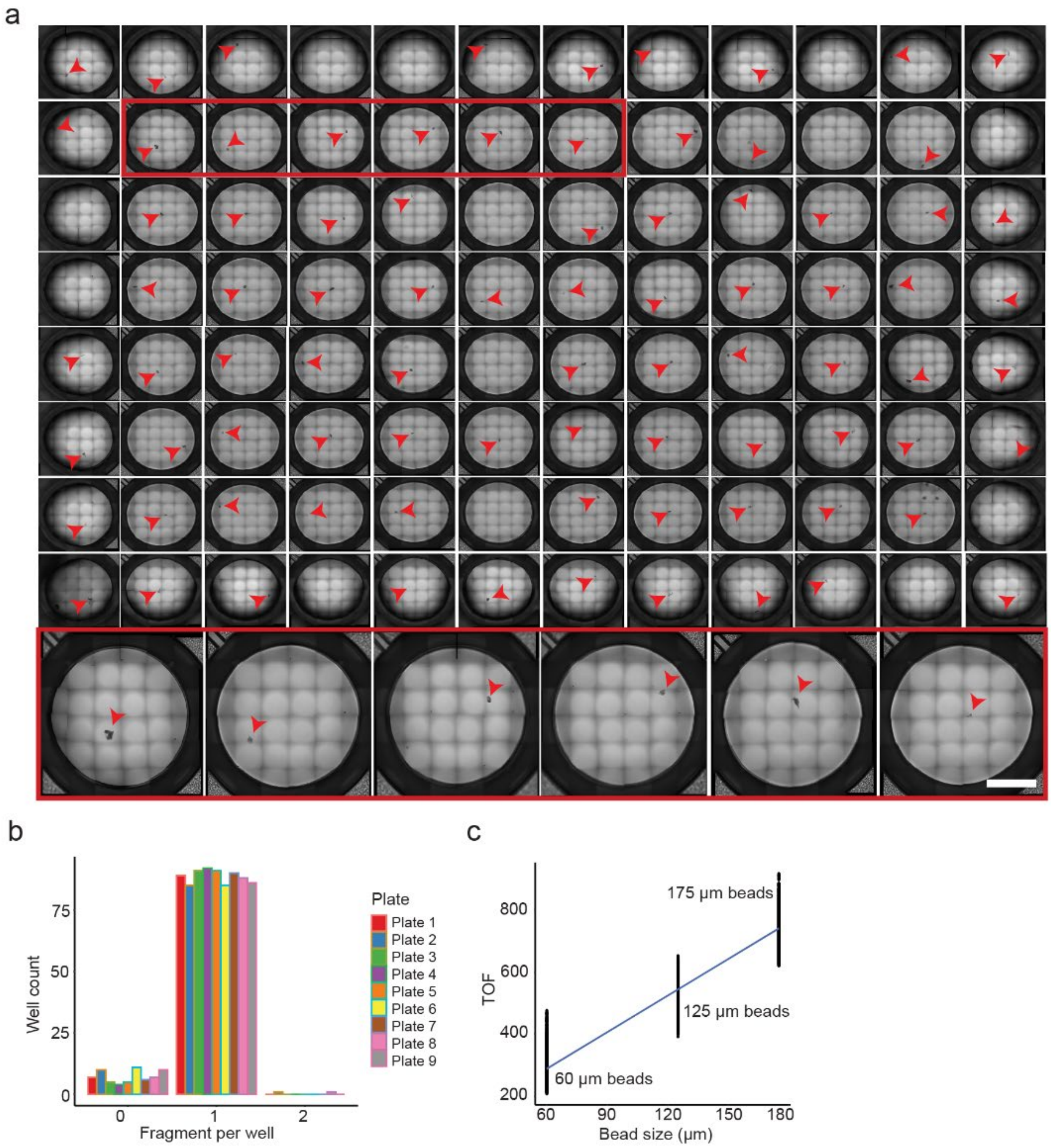

Supplementary Fig. 1: Size-gated sorting of fragments with a large fragment sorter. a, Representative brightfield microscopy image of a 96-well plate containing individually sorted liver fragments. Arrowheads indicate individual fragments. Shown is 1 plate out of 10 from 1 experiment. Scale bar 2 mm. **b**, Barplot showing the number of wells with zero, one, or two fragments. Colors indicate different plates (n = 9 plates, 0 fragment: 1 fragment: 2 fragment,). **c**, A linear standard curve generated by acquiring standard-sized beads [60 µm (n= 982), 125 µm (n = 736), and 175 µm (n = 923)]. A linear model is fitted to calculate fragment sizes. Black dots represent acquired measurements of individual beads. TOF: time of flight. For **b** and **c,** the source data are provided as a Source Data file.

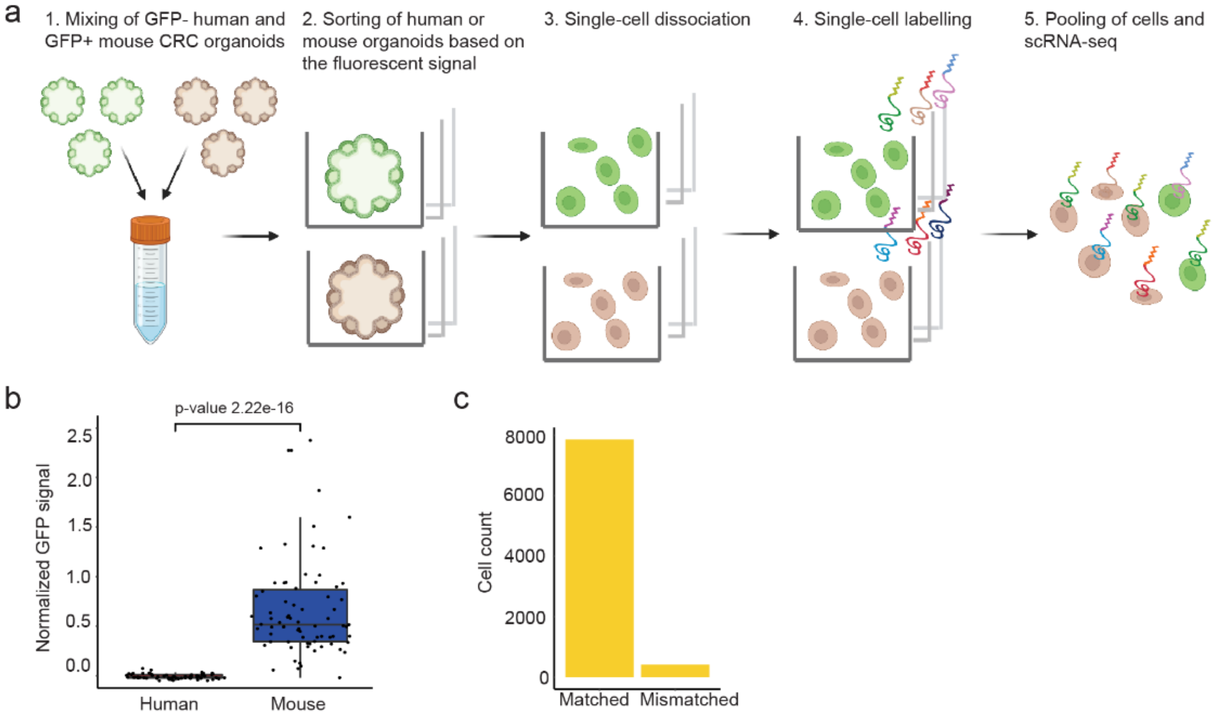

Supplementary Fig. 2: Index-sorting of mixed-species organoids demonstrates the high accuracy of fragment-seq. a, Schematic drawing of experimental workflow. scRNA-seq: single-cell RNA-sequencing. Created with BioRender.com. **b**, Boxplot of normalized GFP signal acquired from sorted GFP- human and GFP+ murine organoids (n = 144 fragments each across 1 sample). Black dots represent individual fragments; the middle line represents the median; the upper and lower lines are the first and third quantiles (Q1 and Q3); the whiskers indicate the upper and lower limits of data spread by subtracting 1.5* interquartile range (IQR) from Q1 and adding 1.5* IQR to Q3. The p-value was calculated with a non-parametric Wilcoxon signed-rank test (two-sided, non-adjusted). **c**, Barplot showing the fraction of cells that were matched or mismatched (n = 1 sample). For **b** and **c,** the source data are provided as a Source Data file.

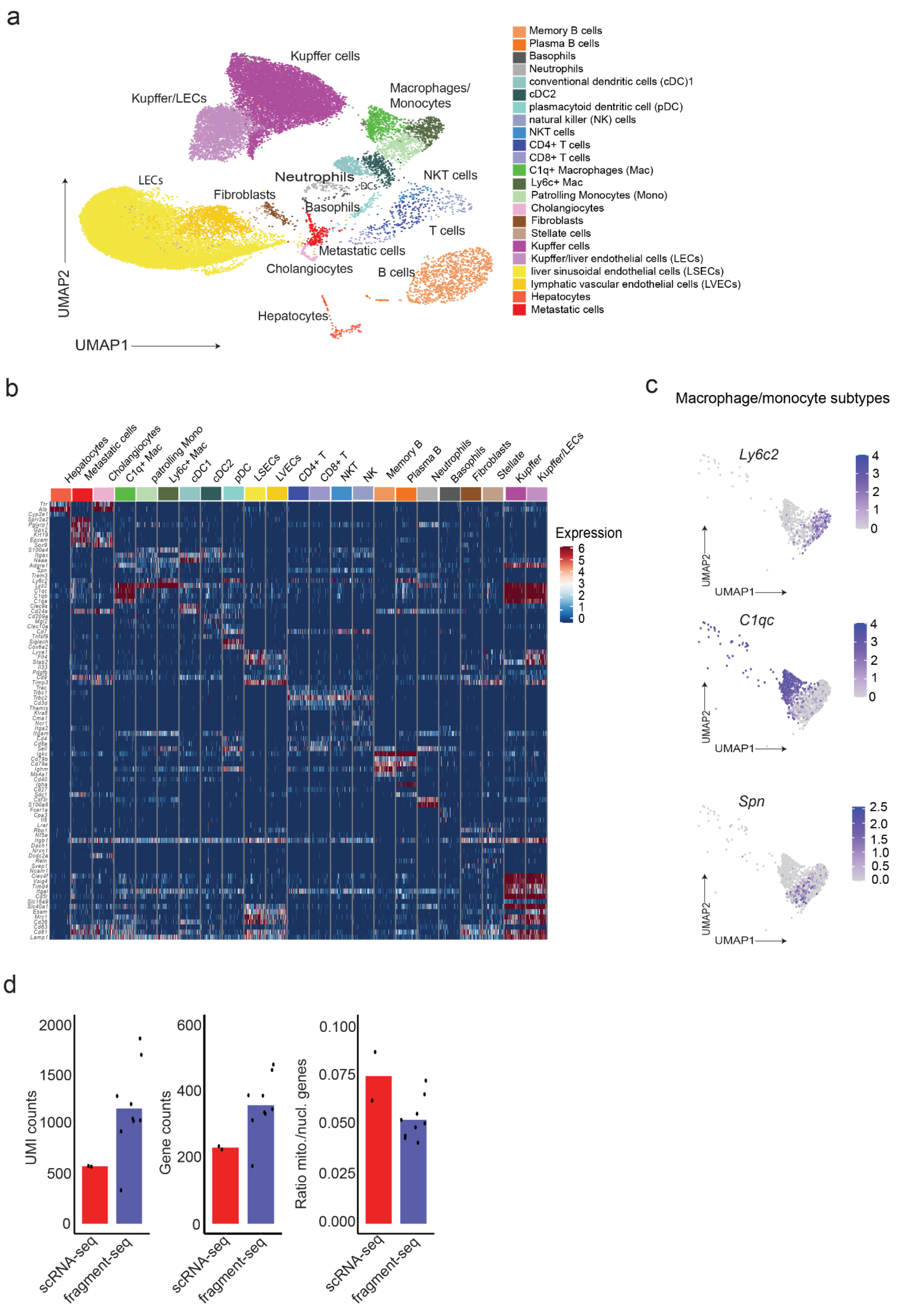

Supplementary Fig. 3: Single-cell annotation of fragment-seq data. a, Uniform Manifold Approximation and Projection (UMAP) visualization of integrated liver fragment-seq samples (n = 10; 9 injected with CRC organoids, 1 untreated). Cells are

clustered, annotated, and colored by their cell type. **b**, Heatmap showing marker genes used for cell type annotation of liver fragment-seq experiments. The columns represent cells and the rows represent genes. Gene expression levels per cell cluster are normalized using a Pearson residual approach. **c**, Feature plots showing expression of marker genes for different subsets in monocytes of fragment-seq liver data. Upper plot, *Ly6c2* indicating *Ly6c*+ macrophages; middle plot, *C1qc* indicating *C1q*+ macrophages; lower plot, *Spn* indicating patrolling monocytes. **d**, Barplots comparing median UMI counts (left), gene counts (middle), and the ratio of mitochondrial to nuclear genes (right) between conventional scRNA-seq and fragment-seq (n = 2 samples for conventional scRNA-seq and 9 samples for fragment-seq). Dots represent individual samples. The upper bar limit shows the mean across samples. Reads were downsampled to 30,000 reads/cell. UMI: Unique molecular identifiers, mito: mitochondrial, nucl: nuclear, scRNA-seq, NS: not significant. For **b** and **d** the source data are provided as a Source Data file.

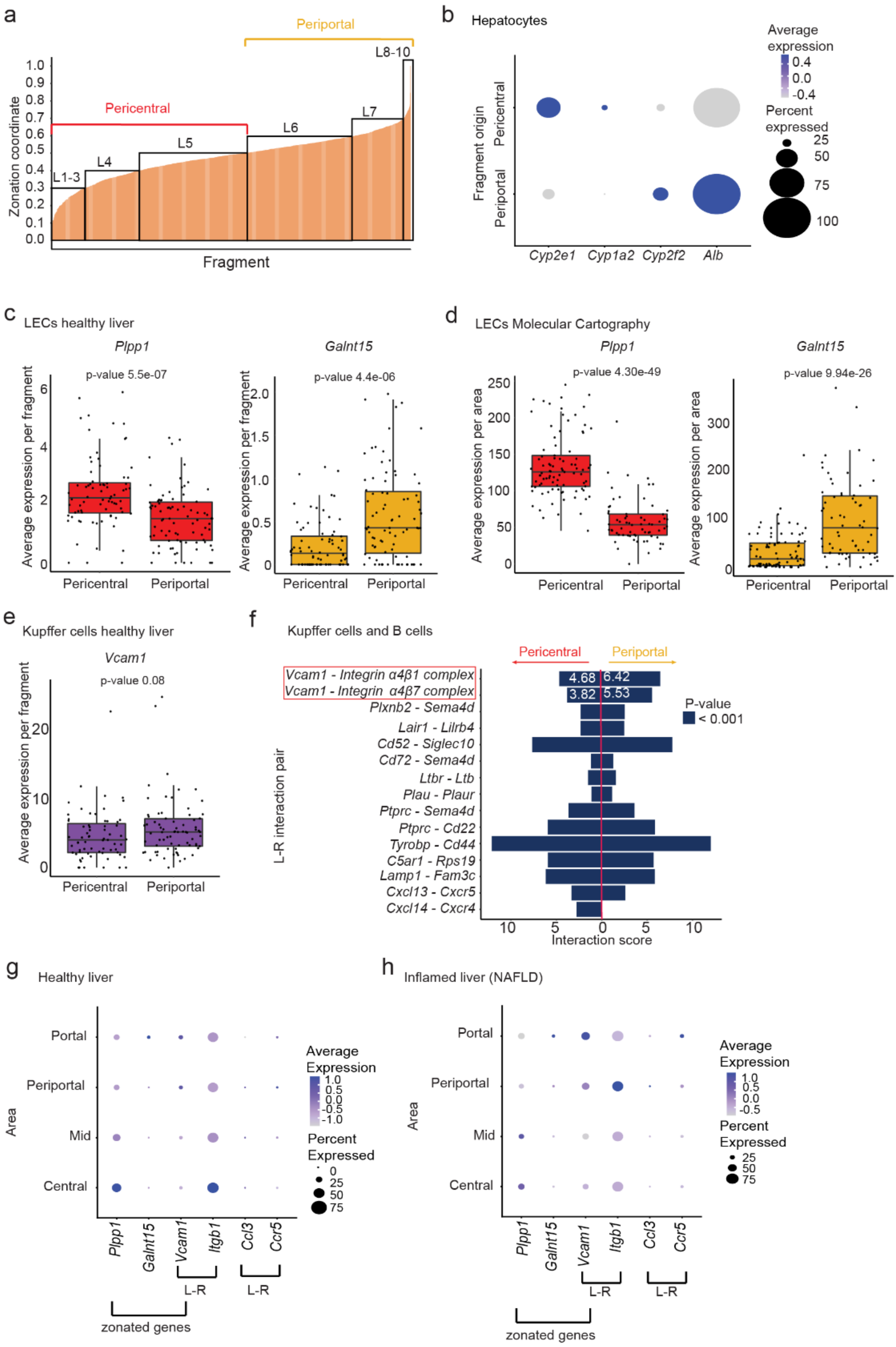
a
Periportal
Pericentral
L8-10
L7
L6
L5
L4
L1-3
Zonation coordinate
1.0
0.9
0.8
0.7
0.6
0.5
0.4
0.3
0.2
0.1
0.0
Fragment
b
Hepatocytes
Average expression
0.4
0.0
-0.4
Percent expressed
25
50
75
100
Fragment origin
Pericentral
Periportal
Cyp2e1
Cyp1a2
Cyp2f2
Alb
c
LECs healthy liver
Plpp1
p-value 5.5e-07
Galnt15
p-value 4.4e-06
Average expression per fragment
Pericentral
Periportal
d
LECs Molecular Cartography
Plpp1
p-value 4.30e-49
Galnt15
p-value 9.94e-26
Average expression per area
Pericentral
Periportal
e
Kupffer cells healthy liver
Vcam1
p-value 0.08
Average expression per fragment
Pericentral
Periportal
f
Kupffer cells and B cells
Pericentral
Periportal
Vcam1 - Integrin α4β1 complex
Vcam1 - Integrin α4β7 complex
Plxnb2 - Sema4d
Lair1 - Lilrb4
Cd52 - Siglec10
Cd72 - Sema4d
Ltbr - Ltb
Plau - Plaur
Ptprc - Sema4d
Ptprc - Cd22
Tyrobp - Cd44
C5ar1 - Rps19
Lamp1 - Fam3c
Cxcl13 - Cxcr5
Cxcl14 - Cxcr4
4.68
6.42
3.82
5.53
P-value
< 0.001
L-R interaction pair
Interaction score
g
Healthy liver
Portal
Periportal
Mid
Central
Area
Average Expression
1.0
0.5
0.0
-0.5
-1.0
Percent Expressed
0
25
50
75
Plpp1
Galnt15
Vcam1
Itgb1
Ccl3
Ccr5
L-R
L-R
zonated genes
h
Inflamed liver (NAFLD)
Portal
Periportal
Mid
Central
Area
Average Expression
1.0
0.5
0.0
-0.5
Percent Expressed
25
50
75
Plpp1
Galnt15
Vcam1
Itgb1
Ccl3
Ccr5
L-R
L-R
zonated genes

Supplementary Fig. 4: Fragment-sequencing application to mouse liver zonation. a, Barplot showing the distribution of zonation coordinates of fragments across different lobule layers and zones (n = L1-3: 143, L4: 243, L5: 452, L6: 478, L7: 207, L8-10: 29 fragments across 10 samples). **b**, Dot plot showing hepatocyte landmark genes of hepatocytes from grouped fragments in pericentral zones: *Cyp2e1, Cyp1a2* or periportal zones: *Cyp2f2, Alb* (pericentral n= 46, periportal n=31 fragments across 10 samples). **c**, Boxplots of zonated genes in liver endothelial cells (LECs) of spatially ordered fragments from the healthy liver (pericentral n = 76, periportal n= 83 fragments across 1 sample). Black dots denote fragments. **d**, Boxplots of *Plpp1* (left) and *Galnt15* (right) in LECs of spatially grouped areas of Molecular Cartography data (pericentral n= 89; periportal n=66 areas across 4 samples). Black dots indicate individual spatial areas. **e**, Boxplots of *Vcam1* in Kupffer cells (KCs) of spatially ordered fragments from the healthy liver (pericentral n = 67; periportal n= 74 across 1 sample). Black dots indicate individual fragments. **f**, Predicted ligand-receptor (L-R) interactions between KCs and B cells in pericentral or periportal zones (n=9 samples). Interaction scores were calculated from fragment-seq data by CellPhoneDB (unadjusted *p* values). For **c** and **e** a non-parametric Wilcoxon signed-rank test (two-sided, non-adjusted) was applied (not significant [NS] > 0.05). **g**, **h**, Dot plots showing average expression of zonated genes, ligands, and receptors in publicly available Visium data from healthy mouse livers(Guilliams et al. 2022) (**g**) and non-alcoholic fatty liver disease (NAFLD) mouse liver(Guilliams et al. 2022) (**h**). For **d** a negative binomial generalized log-linear model, which employs a (two-sided) empirical Bayes quasi-likelihood F-test, was used for statistical testing and *p*-values (Benjamini-Hochberg adjusted) <0.05 were considered significant. For **c, d,** and **e** the middle line represents the median; the upper and lower lines are the first and third quantiles (Q1 and Q3); the whiskers indicate the upper and lower limits of data spread by subtracting 1.5* interquartile range (IQR) from Q1 and adding 1.5* IQR to Q3. For **a-h,** the source data are provided as a Source Data file. L-R: ligand-receptor.

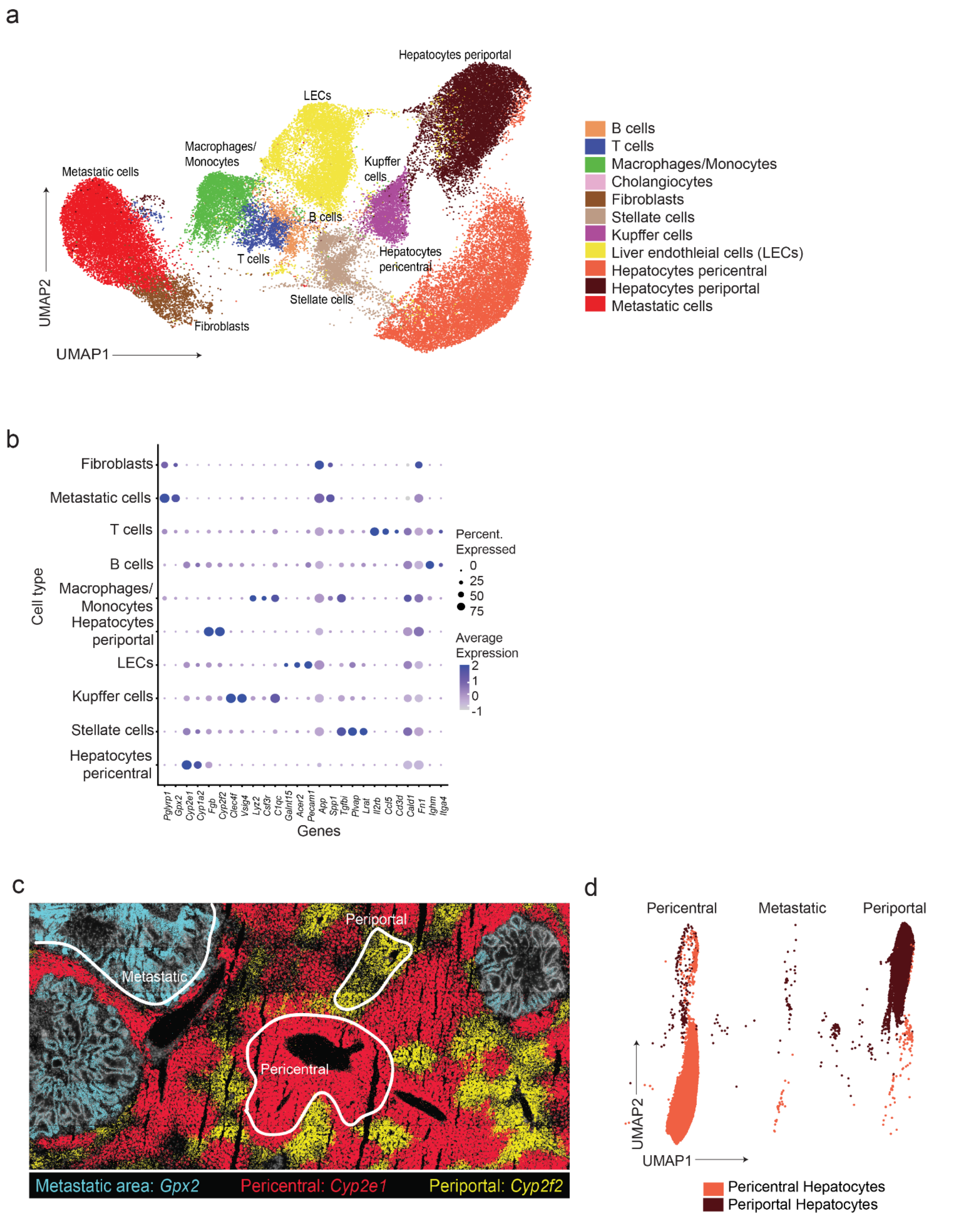

Supplementary Fig. 5: Single-cell annotation of Molecular Cartography data. a, Uniform Manifold Approximation and Projection (UMAP) visualization of single cells after cell segmentation (n = 4 samples). Cells are clustered, annotated, and colored by their cell type. **b**, Dotplot showing marker genes used for cell type annotation. **c**, Representative example of Molecular Cartography of indicated genes for periportal (*Cyp2f2*, yellow), pericentral (*Cyp2e1*, red), and metastatic (*Gpx2*, blue) areas overlayed over DAPI signal (white). Bold white lines denote an example of manually drawn areas grouping cells into different spatial zones. **d**, UMAP visualization of hepatocytes from different spatial zones. Cells are grouped and annotated based on their hepatocyte affiliation; hepatocytes are found in periportal and pericentral areas in dark red and light red, respectively. For **b** the source data are provided as a Source Data file.

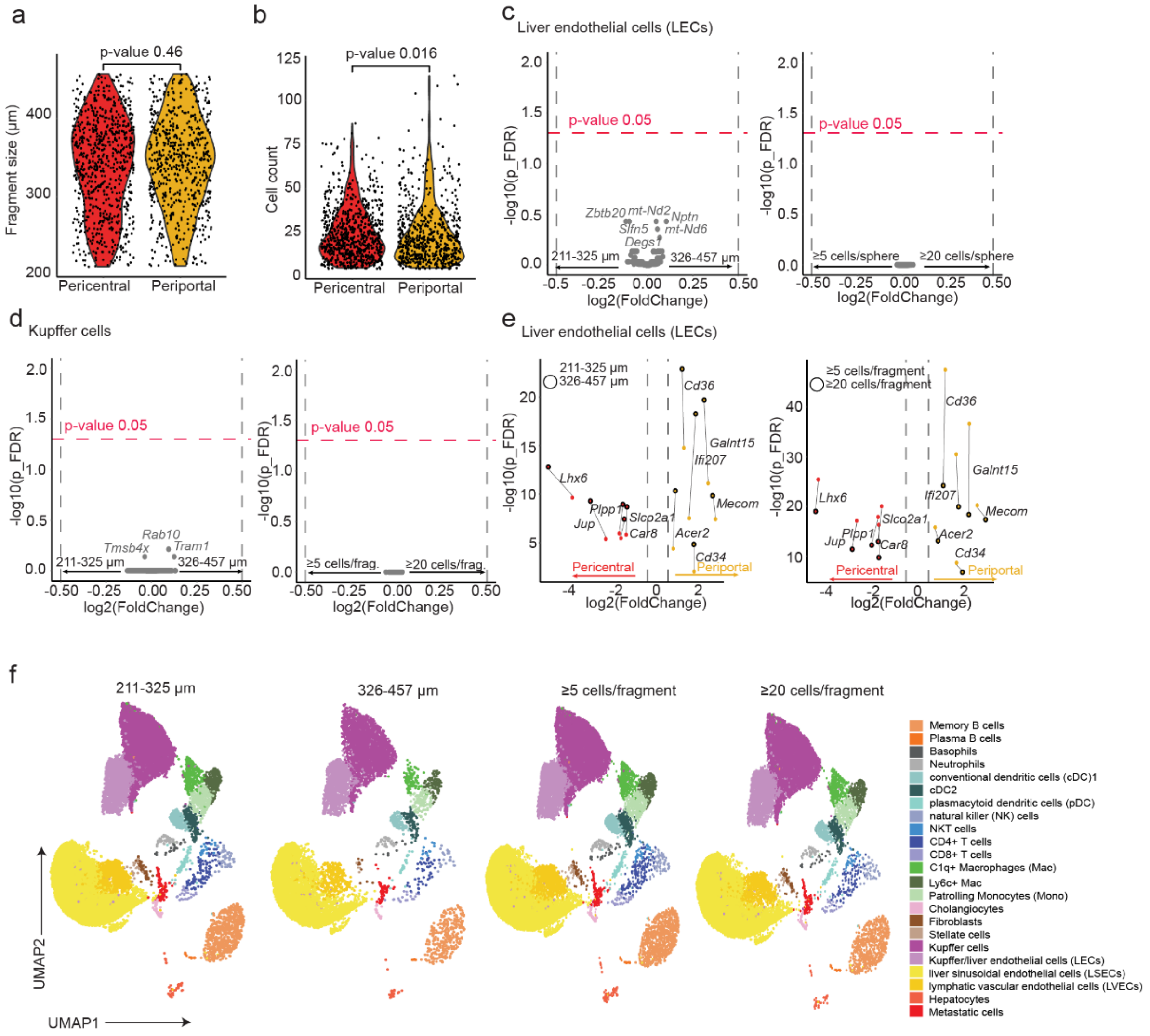

Supplementary Fig. 6: Assessment of biases introduced by fragment size and cell counts per fragment. **a** and **b**, Violin plots comparing fragment sizes (**a**) and cell counts per fragment (**b**) between different lobule areas (n=pericentral: 829, periportal: 714 fragments across 9 samples). *P*-values were calculated with a non-parametric Wilcoxon signed-rank test (two-sided, non-adjusted). **c** and **d**, Differentially expressed genes (DEGs) of liver endothelial cells (LECs) (**c**) and Kupffer cells (KCs) (**d**) comparing different fragment sizes [LECs:211-325 (n=495 fragments) and 326-457µm (n=814 fragments); ≥5 cells/fragment (n=1`543 fragments) and ≥20 cells/fragment (n=800 fragments), KCs:211-325 (n=442 fragments) and 326-457µm (n=778 fragment); ≥5 cells/fragment (n=1`436 fragments) and ≥20 cells/fragment (n=794)]. **e**, Volcano plots of DEGs of LECs in fragments of different sizes (left) or cellularity cutoffs (right) [211-325 (n=495µm fragments), 326-457µm (n=814 fragments), ≥5 cells/fragment (n=1543 fragments), ≥20 cells/fragment (n=800 fragments)]. Colored dots represent significantly enriched genes; red, enriched in pericentral; yellow, enriched in periportal. Gene labels indicate genes significantly enriched in both analyses. Dots from 326-457µm and ≥20 cells/fragment analysis are highlighted by borders. Lines connect the same genes from different analyses. **f**, Uniform Manifold Approximation and Projection (UMAP) from different technical cutoffs (211-325µm: n=9`499, 326-457µm: n=22`673, ≥5 cells/fragment: n=37`024, ≥20 cells/fragment: n=27`991cells). **c**, **d**, and **e,** A negative binomial generalized log-linear model (`glmQLFTest` function of edgeR), which uses a (two-sided) empirical Bayes quasi-likelihood F-test, was used taking lobule layers and batch (**c** and **d**) or batch only (**e**) into account. *P*-values (Benjamini-Hochberg adjusted) <0.05 were considered significant (colored dots). Non-significant (NS, grey dots). p_FDR: false discovery rate adjusted p-value. For **a-e,** the source data are provided as a Source Data file.

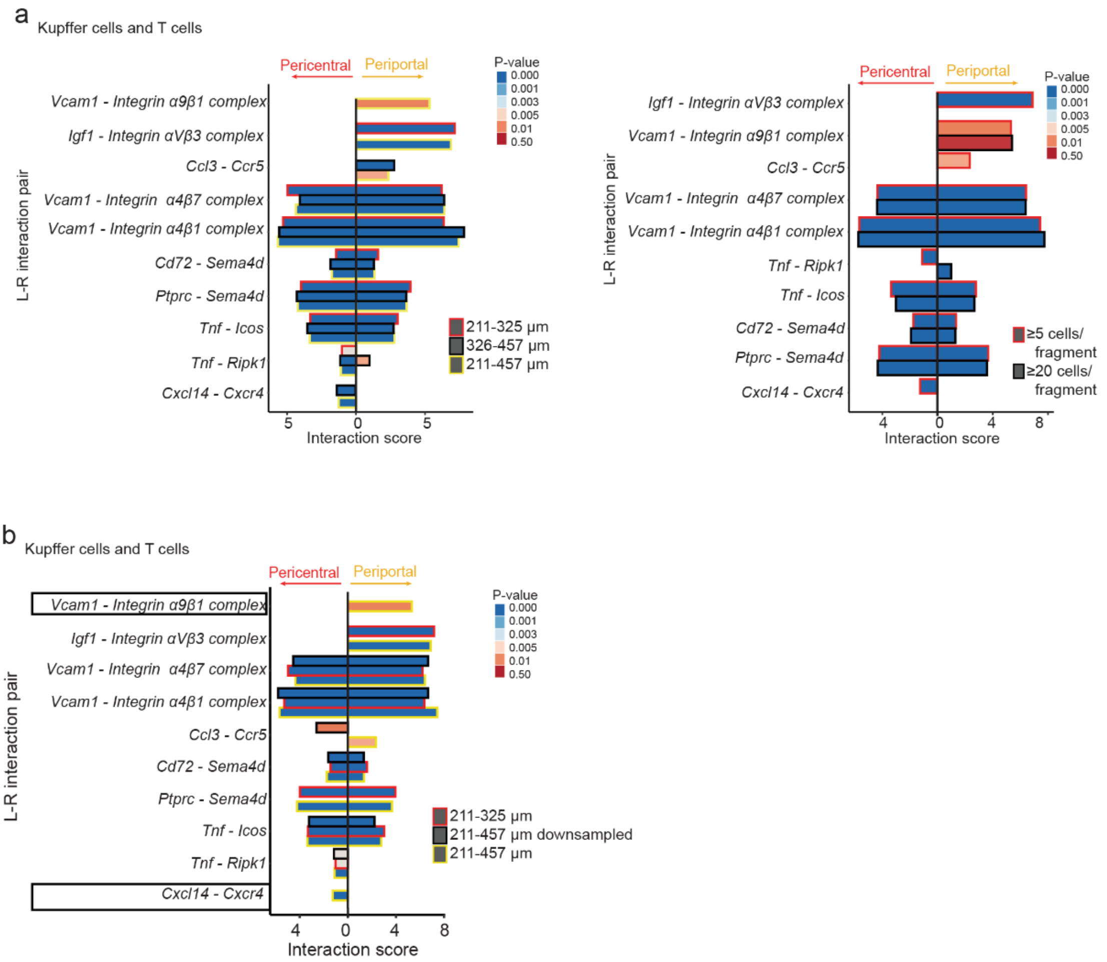

Supplementary Fig. 7: Assessment of biases in ligand-receptor interactions introduced by fragment size and cell counts per fragment.

a,**b,** Predicted ligand-receptor (L-R) interactions between KCs and T cells in pericentral or periportal zones (n=9 samples) calculated by CellPhoneDB using a permutation test (unadjusted p-value indicated). Bar border colors indicate groups in **a** (red: 211-325µm and ≥5 cells/fragment, black: 326-457µm and ≥20 cells/fragment, yellow: 211-457µm) and **b** (red: 211-325µm, black: 211-457µm downsampled to match the cell numbers of the 211-325µm datasets, yellow: 211-457µm). Interactions mentioned in the main text are highlighted with boxes. For **a** and **b** the source data are provided as a Source Data file.

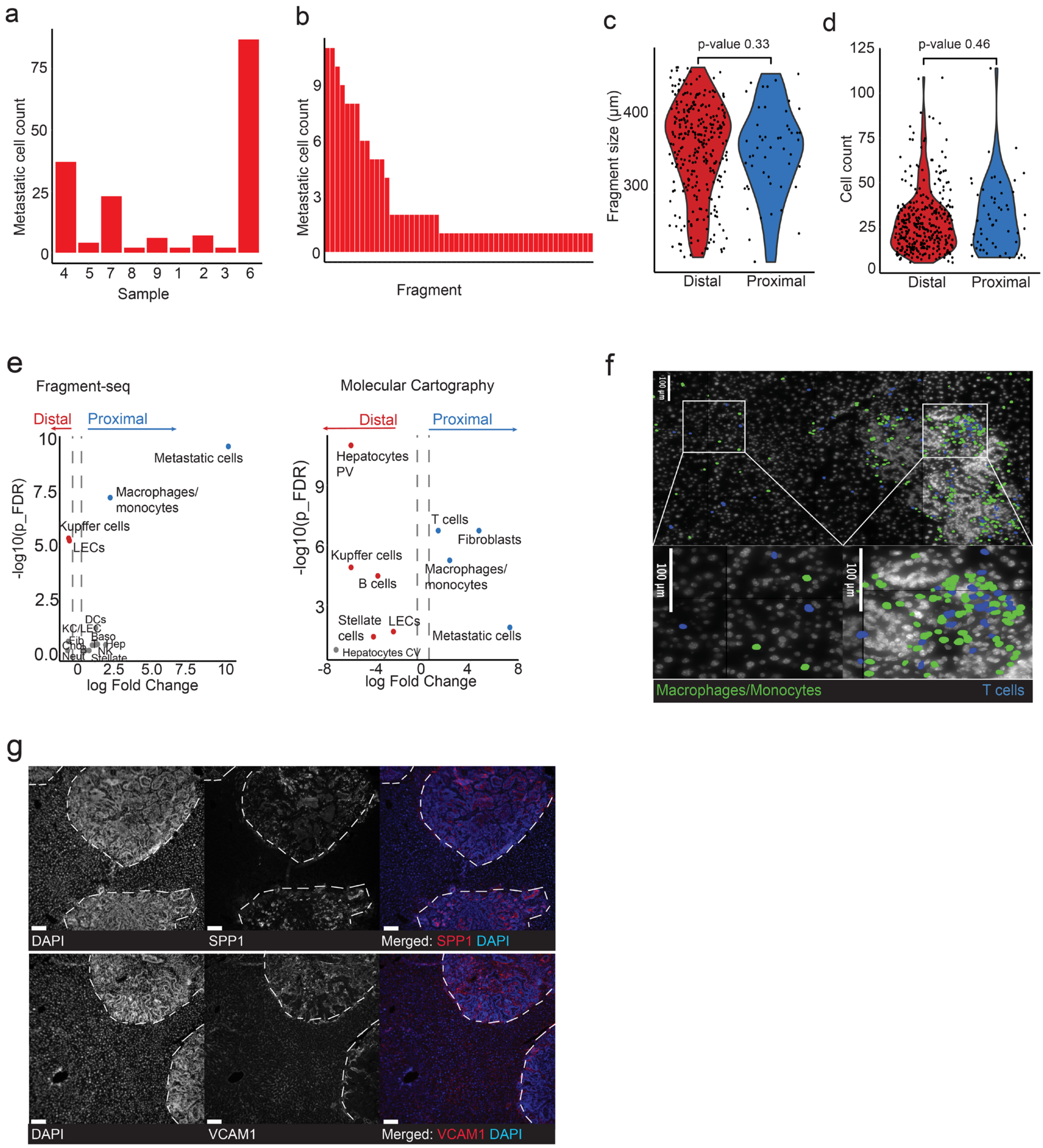

Supplementary Fig. 8: Fragment-sequencing application to mouse metastatic liver. a, Barplot showing the count of metastatic cells within different liver samples (n = 9 samples). **b**, Barplot showing the metastatic cell count per metastatic-proximal fragments (n=54 fragments across 3 samples). **c** and **d**, Violin plots comparing fragment sizes per fragment (**c**) and cell count per fragment (**d**) between distal and proximal areas (distal: 289; n = proximal: 51 fragments across 3 samples). *P*-values were calculated with a non-parametric Wilcoxon signed-rank test (two-sided, non-adjusted). **e**, Differential abundance analysis (DAA) of cell types between areas distal or proximal to metastases in fragment-seq data (left, n = 3 samples) and Molecular Cartography (MC) (right, n= distal: 71 proximal: 11 areas across 2 samples). Colored dots represent significantly higher proportions of cell types; red is higher in distal; blue is higher in proximal. A negative binomial generalized log-linear model was used for statistical testing and *p* values (Benjamini-Hochberg adjusted) <0.05 were considered significant. **f**, MC images with monocytes highlighted in green and T cells in blue comparing distal and proximal metastatic sites overlayed over DAPI signal (white). **g**, Representative immuno-fluorescence images of SPP1 and VCAM1 comparing proximal and distal regions. The dashed line represents the metastatic border region. The scale bar on the left bottom represents 100 µm. NS: not significant. Images in f and g are representative for MC dataset analyzed in e (total of n= distal: 71 proximal: 11 areas across 2 samples). For **a-e,** the source data are provided as a Source Data file. p_FDR: false discovery rate adjusted p-value, DCs: dendritic cells, KC: Kupffer cells, LEC: liver endothelial cell, Neut: Neutrophil, Baso: basophil, NK: natural killer cell, Chol: Cholangiocyte, Fib: Fibroblast, CV: pericentral, PV: periportal.

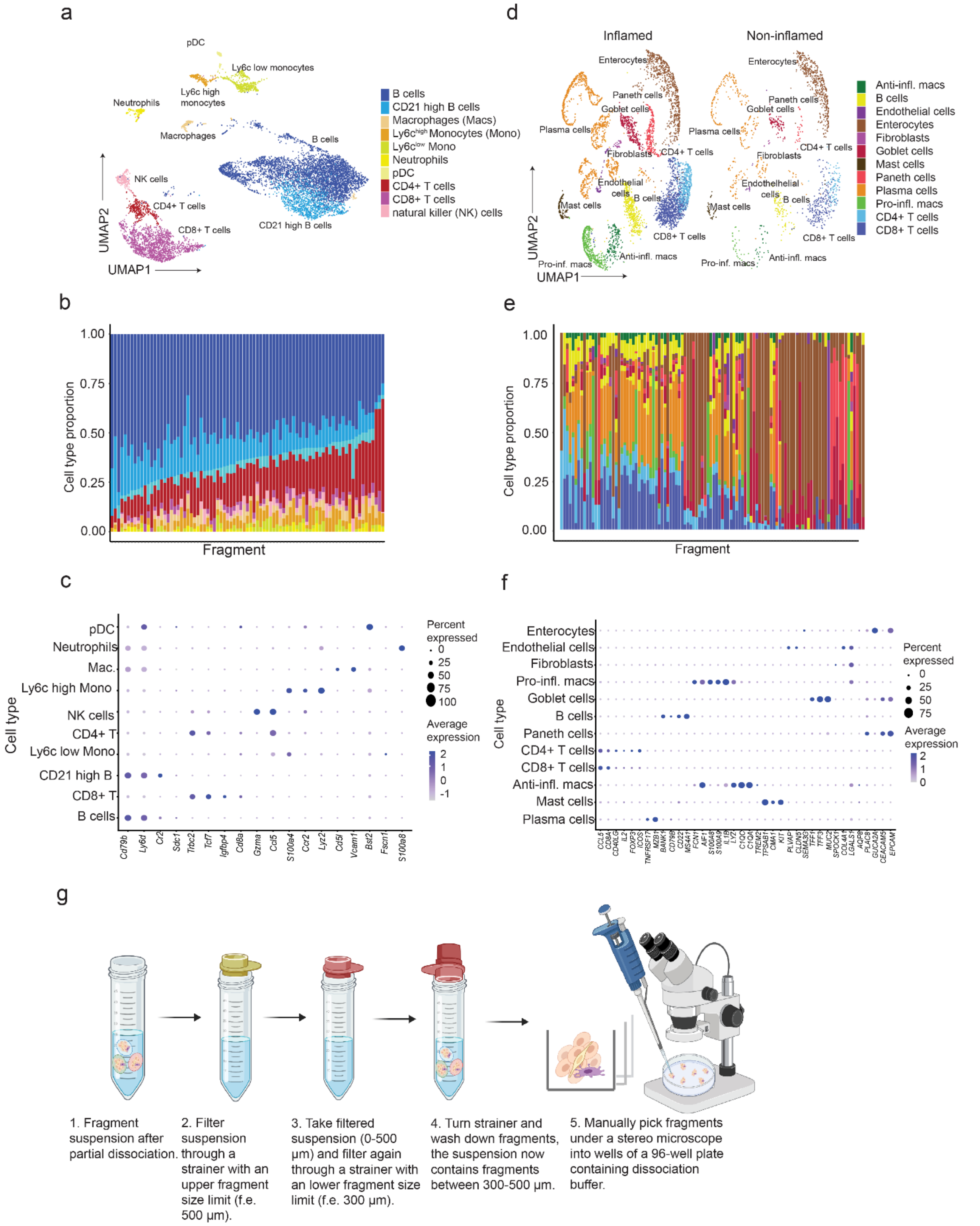

Supplementary Fig. 9: Proof-of-concept of applying fragment-sequencing to other tissues and species. a, Uniform Manifold Approximation and Projection (UMAP) visualization of integrated fragment-seq data of mouse wild-type spleen (n = 2); only fragments with at least 5 cells are considered. Cells are clustered, annotated, and colored according to their cell type. **b**, Barplot showing cell type proportions per fragment (n = 83 fragments across 2 samples). Colors as indicated in a. **c**, Dotplot showing the average gene expression of cell type markers of annotated clusters. **d**,**e,** and **f**, Same visualization as in **a**, **b,** and **c** but for Crohn's disease biopsy samples (n = 62 fragments across 2 samples). **g**, Schematic drawing of size selection of fragments for manual picking instead of using the large fragment sorter (Biorender.com). pDC: plasmacytoid dendritic cell. Created with BioRender.com. For **b**,**c**, **e**, and **f** the source data are provided as a Source Data file.

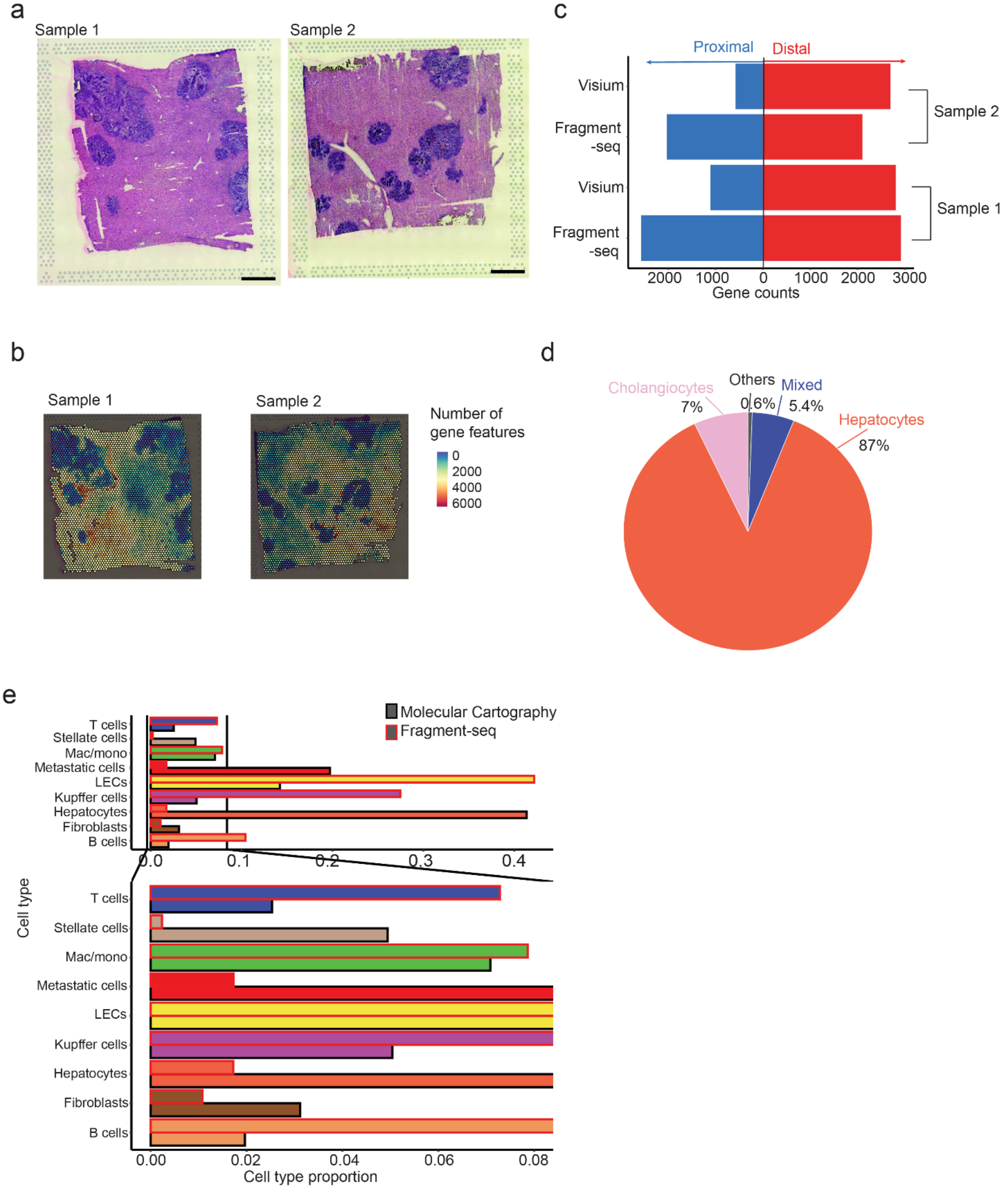

Supplementary Fig. 10: Comparison of fragment-sequencing with Visium and Molecular Cartography. a, Hematoxylin and eosin (H&E) staining of mouse metastatic livers (n = 2). Metastatic areas are indicated by dark purple stains. Scale bars 1 mm. **b**, Number of gene features per spot projected on H&E stainings from a. **c**, Boxplot comparing the number of features from different spatial areas of fragment-seq to Visium (n = 2 each). The same two samples were used for fragment-seq and Visium, indicated by brackets. **d**, Pie Chart indicating the average proportions of cell types after spot deconvolution of integrated mouse metastatic liver samples (n=2). Spots were assigned to one single-cell type if at least 75% of transcripts could be assigned to that cell type; other spots were annotated as mixed. **e**, Zoomed-in barplot comparing the cell type proportions between Molecular Cartography (black borders) and fragment-seq (red borders) of cell types that could be robustly detected in both datasets. For **c-e** the source data are provided as a Source Data file.

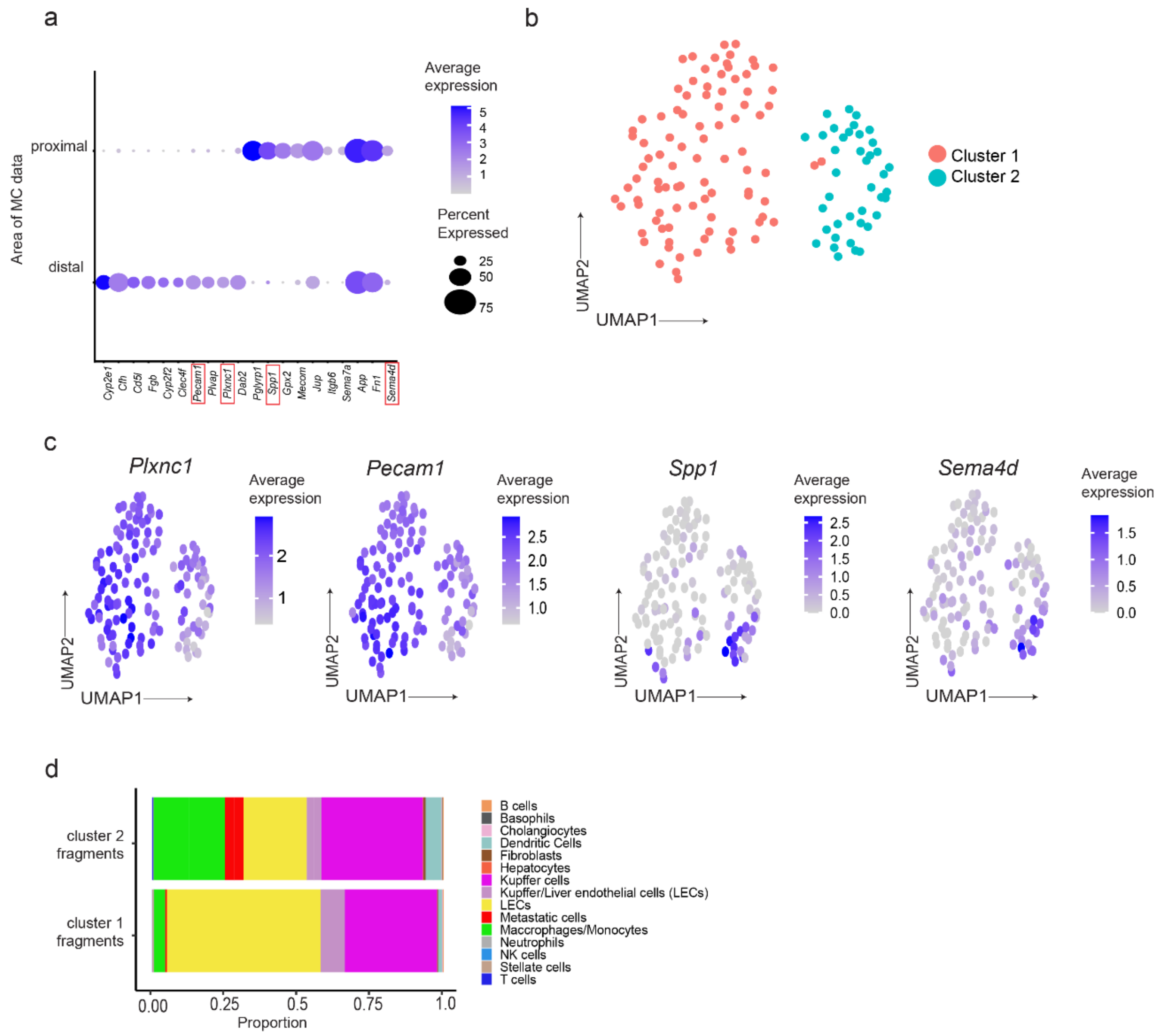

Supplementary Fig. 11: Pseudobulk clustering with spatially defined marker genes to locate fragment position: a, Dot plot showing top ten differentially expressed genes of proximal compared to distal areas (and vice versa) of Molecular Cartography (MC) data. (distal n = 155, proximal n= 11 areas across 2 samples). Red rectangles indicate markers also shown in **c**. **b,** Uniform Manifold Approximation and Projection (UMAP) visualization of pseudobulks of fragments (n = 133 fragments across one sample) using 10 principal components built from only the 20 markers identified in **a. c,** Feature plots showing expression of four examples of marker genes used for pseudobulk generation of fragment-seq liver data. Starting from the left: first, *Plxnc1*; second, *Pecam1*; third, *Spp1*; fourth, *Sema4d*. **d,** Barplot showing cell type proportions per cluster. **d**, Barplot showing cell type proportions per cluster. For **a** and **d** the source data are provided as a Source Data file.

Supplementary References

Guilliams, Martin, Johnny Bonnardel, Birthe Haest, Bart Vanderborght, Camille Wagner, Anneleen Remmerie, Anna Bujko, et al. 2022. "Spatial Proteogenomics Reveals Distinct and Evolutionarily Conserved Hepatic Macrophage Niches." *Cell* 185 (2): 379–96.e38.
