# Supplementary material for: Fragment-sequencing unveils local tissue microenvironments at single-cell resolution
Source: Nat Commun. 2023 Nov 27;14:7775. doi: 10.1038/s41467-023-43005-8 (PMC10681997; doi:10.1038/s41467-023-43005-8)
Supplement: Supplementary file 3 — Description of Additional Supplementary Files [file 41467_2023_43005_MOESM3_ESM.pdf]

<u>Description of Additional Supplementary Files</u>

Supplementary Data 1: MULTI-seq_3Prime_sequences: This file contains the primer sequences used for labeling of cells following the MULTI-seq method and a 3’ scRNA-seq capture approach. The first column shows the well position within 3x96 well plates; the second column shows the name of the barcode; the third column represents the barcode sequence and the fourth column shows the oligo sequence which contains a primer sequence for lipid anchor binding and amplification (CCTTGGCACCCGAGAATTCCA), an 8 bp barcode sequence and a poly-A stretch for single-cell RNA-seq capture.

Supplementary Data 2: MULTI-seq_5Prime_sequences: This file contains the primer sequences used for labeling of cells following the MULTI-seq method and a 5’ scRNA-seq capture approach. The first column shows the well position within 2x96 well plates; the second column shows the name of the barcode; the third column represents the barcode sequence and the forth column shows the oligo sequence which contains a primer sequence for lipid anchor binding and amplification (CCTTGGCACCCGAGAATTCCA), an 8 bp barcode sequence and a 5’ capture sequence (CCCATATAAGAAA).
