# Supplementary material for: Fragment-sequencing unveils local tissue microenvironments at single-cell resolution
Source: Nat Commun. 2023 Nov 27;14:7775. doi: 10.1038/s41467-023-43005-8 (PMC10681997; doi:10.1038/s41467-023-43005-8)
Supplement: Supplementary file 6 — Reporting Summary [file 41467_2023_43005_MOESM6_ESM.pdf]

nature portfolio

Corresponding author(s): Andreas E. Moor

Last updated by author(s): 2023.08.17

Reporting Summary

Nature Portfolio wishes to improve the reproducibility of the work that we publish. This form provides structure for consistency and transparency in reporting. For further information on Nature Portfolio policies, see our Editorial Policies and the Editorial Policy Checklist.

Statistics

For all statistical analyses, confirm that the following items are present in the figure legend, table legend, main text, or Methods section.

n/a	Confirmed	
☐	☒	The exact sample size (*n*) for each experimental group/condition, given as a discrete number and unit of measurement
☐	☒	A statement on whether measurements were taken from distinct samples or whether the same sample was measured repeatedly
☐	☒	The statistical test(s) used AND whether they are one- or two-sided<br>*Only common tests should be described solely by name; describe more complex techniques in the Methods section.*
☐	☒	A description of all covariates tested
☐	☒	A description of any assumptions or corrections, such as tests of normality and adjustment for multiple comparisons
☐	☒	A full description of the statistical parameters including central tendency (e.g. means) or other basic estimates (e.g. regression coefficient) AND variation (e.g. standard deviation) or associated estimates of uncertainty (e.g. confidence intervals)
☐	☒	For null hypothesis testing, the test statistic (e.g. *F*, *t*, *r*) with confidence intervals, effect sizes, degrees of freedom and *P* value noted<br>*Give P values as exact values whenever suitable.*
☒	☐	For Bayesian analysis, information on the choice of priors and Markov chain Monte Carlo settings
☒	☐	For hierarchical and complex designs, identification of the appropriate level for tests and full reporting of outcomes
☒	☐	Estimates of effect sizes (e.g. Cohen's *d*, Pearson's *r*), indicating how they were calculated

Our web collection on statistics for biologists contains articles on many of the points above.

Software and code

Policy information about availability of computer code

Data collection	Data in this study was collected with a NovaSeq 6000 system (Illumina) using NovaSeq SP Reagent Kits (100 cycles) v1.5 and S4 Reagent kits (200 cycles) v1.5 with XP workflow. BCL files were demultiplexed using Bcl2fastq (v2.20.0.422). Data collection of Molecular Cartography was done by Resolve BioSciences. Fastq files from BD Rhapsody scRNAseq data were processed using zUMIs (v.2.9.4) and STAR (v2.5.2b) with genecode GRCh38 v2020-A for human samples and gene code GRCm38 vM25 for mouse samples, for the mouse/human organoid mixing experiment a merged genecode from both species and a GFP 3' UTR sequence was used. Chromium 10X scRNAseq data were processed using Cell Ranger (v5.0.0) with GRCm38 v2020-A genecode. Visium data were processed using SpaceRanger (v1.2.0) with GRCm38 v2020-A genecode.
Data analysis	Data analysis was done using R v4.1.0 and Python v3.8.10. The following R pacakges were used: DNAbarcodes (v1.20.0), Seurat (v4.0.3), scran (v1.22.1), SingleCellExperiment (v1.16.0), dplyr10 (v1.0.7), tidyverse11 (v1.3.1), ggplot2 (v3.3.5), biomaRt (v2.50.3), deMULTIplex (v1.0.2), batchelor (v1.10.0), edgeR (v3.36.0), celda (v1.12.0), SCDC (v0.0.0.9000), RANN (v2.6.1), igraph (v1.3.4). The following python packages were used: CellPhoneDB (v4.0.0), Cellpose (v2.0.4). For Molecular Cartography image analysis, we used ImageJ2 (v2.9.0) with the Polylux plugin (v1.9.0).

For manuscripts utilizing custom algorithms or software that are central to the research but not yet described in published literature, software must be made available to editors and reviewers. We strongly encourage code deposition in a community repository (e.g. GitHub). See the Nature Portfolio guidelines for submitting code & software for further information.

Data

Policy information about availability of data

All manuscripts must include a data availability statement. This statement should provide the following information, where applicable:

- Accession codes, unique identifiers, or web links for publicly available datasets
- A description of any restrictions on data availability
- For clinical datasets or third party data, please ensure that the statement adheres to our policy

The data generated in this study (gene expression data for human fragment-seq, RNA-sequencing data for murine scRNA-seq and fragment-seq, and Visium data) have been deposited in the Gene Expression Omnibus (GEO) database with accessory number GSE216189 [https://www.ncbi.nlm.nih.gov/geo/query/acc.cgi?acc=GSE216189]. The Molecular Cartography data from Resolve are deposited at Zenodo with the accession number 8413573 [https://zenodo.org/record/8413573]. The raw RNA-sequencing data for Crohns' Disease biopsies are protected and are not available due to Swiss ethics regulations and data privacy laws. The processed gene expression data from patients are included in the GSE216189 dataset. Data generate in this study on fragment sizes, UMI counts, GFP signal and cell counts of species mixing experiments, cell type proportion in fragments, enriched genes in different hepatic zones and/or cell types, L-R interactions, colocalization scores, fragments sorted per cell, TOF of beads, UMI counts, gene counts and percentage of mitochondrial genes in scRNA-seq and fragment-seq, analyses with different fragment size cutoffs, fragment size and cellularity, number of metastatic cells per sample and fragment, cell type enrichment in fragment-seq, MC, and Visium as well as Visium gene counts are provided in the Source Data file.
The previously published data used in this study from the liver cell atlas (Guilliams et al., 2022) are available in the GEO database under accession number GSE192742 [https://www.ncbi.nlm.nih.gov/geo/query/acc.cgi?acc=GSE192742].

Human research participants

Policy information about studies involving human research participants and Sex and Gender in Research.

Reporting on sex and gender	Sex and gender were not included in the study design, as these factors are not relevant for the proof-of-principle that Fragment-seq can be applied to human samples. The research team carried out experiments with coded specimens, and has no access to further patient information due to data privacy and ethic regulations.
Population characteristics	The research team carried out experiments with coded specimens, and has no access to further patient information due to data privacy and ethic regulations.
Recruitment	Recruitment was carried our by the team of Prof. Dr. Scharl at the University Hospital Zurich in accordance with procedures approved by the Cantonal Ethics Committee. Written informed consent was collected before study inclusion from all participants.
Ethics oversight	Ethical approval for collecting tissue specimen from IBD patients was received by the Cantonal Ethics Committee of the Canton Zürich (BASEC-No.: EK-1755/ PB_2019-00169).

Note that full information on the approval of the study protocol must also be provided in the manuscript.

Field-specific reporting

Please select the one below that is the best fit for your research. If you are not sure, read the appropriate sections before making your selection.

☒ Life sciences ☐ Behavioural & social sciences ☐ Ecological, evolutionary & environmental sciences

For a reference copy of the document with all sections, see nature.com/documents/nr-reporting-summary-flat.pdf

Life sciences study design

All studies must disclose on these points even when the disclosure is negative.

Sample size	No statistical method was used to predetermine sample size, instead, sample sizes were chosen based on comparable studies in the field (for example paired cell sequencing (Halpern et al. 2018) n=3, PIC-seq n=4-7 (Giladi et al. 2020), Clump-seq n=3 (Manco et al. 2021)). In addition, biological interpretations were constrained by statistical significance obtained through the given sample size.<br><br>Giladi, Amir, Merav Cohen, Chiara Medaglia, Yael Baran, Baoguo Li, Mor Zada, Pierre Bost, et al. 2020. "Dissecting Cellular Crosstalk by Sequencing Physically Interacting Cells." Nature Biotechnology 38 (5): 629–37.<br>Halpern, Keren Bahar, Rom Shenhav, Hassan Massalha, Beata Toth, Adi Egozi, Efi E. Massasa, Chiara Medgalia, et al. 2018. "Paired-Cell Sequencing Enables Spatial Gene Expression Mapping of Liver Endothelial Cells." Nature Biotechnology 36 (10): 962–70.<br>Manco, Rita, Inna Averbukh, Ziv Porat, Keren Bahar Halpern, Ido Amit, and Shalev Itzkovitz. 2021. "Clump Sequencing Exposes the Spatial Expression Programs of Intestinal Secretory Cells." Nature Communications 12 (1): 3074.
Data exclusions	Fragments with less than 5 cells were filtered out during analyses, as they are not sufficient to represent cellular heterogeneity of a niche. In addition, for analyses centered around distal and proximal fragments we focused on the 3 out of 9 metastases-bearing animals with the

highest tumor burden (at least 20 metastatic cells per sample). Likewise, to assess the differentially expressed genes (DEGs) of macrophages/ monocytes between in distal and proximal regions from MC we used only the 2 of the 4 samples from mice with high metastatic burden (visible macrometastases).

Replication

Fragment-sequencing was replicated with 10 mice for liver, 2 mice for spleen, 2 Crohn's disease biopsies and 1 mixed mouse/human colon cancer organoids samples. All these experiments were successful and showed reproducibility of the fragment-sequencing method. During the process of method development approximately another 10 experiments were performed that were not successful in terms of getting enough cells to process them for single cell RNAseq. For all other types of experiments (i.e. counting fragments per well, single cell RNA-sequencing of the liver, Visium,...) all replicates are shown in the data an indicated in the legends, and we have no failed replicates to report.

Randomization

Fragments were sorted in a random process, but then they were allocated to spatial niches based on landmark gene expression or presence/ absence of certain cell type. Allocation is controlled by testing landmark gene expression in an independent cell type not used for spatial allocation. Experimental animals were not allocated to different treatment groups in this study, therefore, randomization is not relevant to experiments.

Blinding

Blinding is not relevant to this study. We used subjective meassures to assess accuracy or our methods.

Reporting for specific materials, systems and methods

We require information from authors about some types of materials, experimental systems and methods used in many studies. Here, indicate whether each material, system or method listed is relevant to your study. If you are not sure if a list item applies to your research, read the appropriate section before selecting a response.

Materials & experimental systems

n/a	Involved in the study
☐	☒ Antibodies
☐	☒ Eukaryotic cell lines
☒	☐ Palaeontology and archaeology
☐	☒ Animals and other organisms
☒	☐ Clinical data
☒	☐ Dual use research of concern

Methods

n/a	Involved in the study
☒	☐ ChIP-seq
☒	☐ Flow cytometry
☒	☐ MRI-based neuroimaging

Antibodies

Antibodies used

rabbit anti-mouse SPP1: Catalog #MAB808, Clone # 2139B, Lucerna-Chem AG, validated through western blotting by manufacturer, working concentration 5 µg/ml;
goat anti-mouse VCAM1: Catalog #AF643, polyclonal, Lucerna-Chem AG, validated through western blotting by manufacturer, working concentration 5 µg/ml.
cross-absorbed donkey anti-goat Alexa Fluor 647, #A21447, polyclonal, Invitrogen, working concentration 1:400;
cross-absorbed goat anti-rabbit AF647, polyclonal, #A21244, Invitrogen, working concentration 1:400

Validation

Antibodies #MAB808 and #AF643 were validated by manufacturer though western blotting, while secondary antibodies are cross-absorbed.

Eukaryotic cell lines

Policy information about cell lines and Sex and Gender in Research

Cell line source(s)

VilCreERT2;APCfl/fl;Tp53fl/fl;KrasG12D/wt (AKP) organoids: obtained from Owen Sansom from Beatson Institute for Cancer Research in Glasgow.
VilCreERT2;APCfl/fl;Tp53fl/fl; KrasG12D/wt;Smad4KO (AKPS) organoids: modified AKP organoids with an additional knockout in Smad4.
GFP positive VilCreERT2;APCfl/fl;Tp53fl/fl;KrasG12D/wt (AKP) organoids: AKP organoids labeled with the plasmid pMSCV-loxp-dsRed-loxp-eGFP-Ruo-WPRE.
Human colon cancer organoids were obtained from the Visceral Surgery Research Laboratory lead by Salvatore Piscuoglio at University of Basel.

Authentication

The AKP organoids were authenticated by genotyping. The AKPS organoids were selected by addition of TGFB and signaling was checked by qPCR. The GFP AKP organoids were authenticated by analyzing GFP signal with FACS and microscopy. For the human colon cancer organoids we confirmed with scRNAseq that they were human cells, we did not do any other authentication.

Mycoplasma contamination

Organoid lines were tested negative for mycoplasma contamination.

Commonly misidentified lines
(See ICLAC register)

No commonly misidentified lines were used in this study.

Animals and other research organisms

Policy information about studies involving animals; ARRIVE guidelines recommended for reporting animal research, and Sex and Gender in Research

Laboratory animals	Male and female C57BL/6J mice were used aged 6-10 weeks. Animals were housed on a 12 h day–night cycle, with ad libitum access to standard diet and drinking water in a room with controlled temperature (21 ± 2°C) and humidity (55 ± 10%).
Wild animals	This study did not involve wild animals.
Reporting on sex	Sex was not considered a factor in study design, as the fragment-seq method has no gender specific components. Both male and female mice were used. Information on sex has not been collected.
Field-collected samples	This study did not involve field-collected samples.
Ethics oversight	Animal experiments were approved by the Cantonal Veterinary Office Basel-City under the national license number 35370.

Note that full information on the approval of the study protocol must also be provided in the manuscript.
